# ZnO/MO_x_ Nanofiber Heterostructures: MO_x_ Receptor’s Role in Gas Detection

**DOI:** 10.3390/s25020376

**Published:** 2025-01-10

**Authors:** Vadim Platonov, Oleg Sinyashin, Marina Rumyantseva

**Affiliations:** 1 Chemistry Department, Moscow State University, Moscow 119991, Russia; 2 Federal Research Center Kazan Scientific Center RAS, Kazan 420111, Russia

**Keywords:** ZnO nanofibers, surface modification, binding energy of chemisorbed oxygen, heterojunction, band bending, semiconductor gas sensor

## Abstract

ZnO/MO_x_ (M = Fe^III^, Co^II,III^, Ni^II^, Sn^IV^, In^III^, Ga^III^; [M]/([Zn] + [M]) = 15 mol%) nanofiber heterostructures were obtained by co-electrospinning and characterized by X-ray diffraction, scanning electron microscopy and X-ray fluorescence spectroscopy. The sensor properties of ZnO and ZnO/MO_x_ nanofibers were studied toward reducing gases CO (20 ppm), methanol (20 ppm), acetone (20 ppm), and oxidizing gas NO_2_ (1 ppm) in dry air. It was demonstrated that the temperature of the maximum sensor response of ZnO/MO_x_ nanofibers toward reducing gases is primarily influenced by the binding energy of chemisorbed oxygen with the surface of the modifier’s oxides. When detecting oxidizing gas NO_2_, high sensitivity at a low measurement temperature can be achieved with a high concentration of free electrons in the near-surface layer of zinc oxide grains, which is determined by the band bending at the ZnO/MO_x_ interface characterized by the difference in the electron work function of ZnO and MO_x_.

## 1. Introduction

The most common approaches to improve the sensitivity, selectivity, and stability of semiconductor gas sensors are: (i) technological, involving the use of preconcentrators and active or passive filters; (ii) operational, using operating temperature modulation modes or additional measured parameters; and (iii) chemometric, including various data processing algorithms. The fundamental chemical approach is based on the complication of the composition of semiconductor metal oxides forming a sensitive layer, which can be implemented through the introduction of additives providing surface functionalization, bulk doping, and the creation of multiphase nanocomposites combining two or more semiconductor oxides (M^I^O/M^II^O) sensing material. It is known that semiconducting oxides of the *n* and *p*-type of conductivity are characterized by various sensor properties due to differences in the M-O_lat_ [1,2] metal–oxygen bond strength and MO-O_ads_ [3] chemisorbed oxygen bond energy. Depending on the M^I^ and M^II^ nature, M^I^/M^II^ mole ratio, and structural features as well as M^I^O and M^II^O electrical behavior, the formation of different types of nanocomposites is possible: (i) a solid solution (M^I^O)_x_(M^II^O)_1−x_ due to the mutual diffusion of components, (ii) a structure with a sharp *n-n* or *n-p* barriers in the heterojunction region, and (iii) a structure including complex phases M^I^M^II^O_y_ appearing at the interface as a result of the chemical interaction of the M^I^O and M^II^O metal oxides [4,5,6,7,8,9].

The review article [10] considers the concepts that are currently used to explain the mechanisms by which the effect of additives on the characteristics of semiconductor materials formed by oxide nanoparticles arranged in a porous layer is realized. The authors discuss various chemical and/or electrical factors that ensure a change in the sensitivity and selectivity of semiconductor materials, mainly in cases where noble metal nanoparticles act as additives. Of course, the concepts presented can generally be applied to combinations of semiconductor metal oxides and a wide range of additives. However, because they possess different adsorption capabilities and reactivities when interacting with gases, the components of the composite can significantly affect the complex acid–base and oxidation–reduction processes, which determine the sensitivity and selectivity of a sensing material [2]. There are some reviews concerning the sensor properties of composites or heterostructures based on metal oxides [11,12,13,14,15]. However, the difference in the metal oxide nature and morphology, methods of synthesis, and detected gases does not allow us to represent the regularities of the structure and sensor properties of M^I^O/M^II^O materials.

This work presents the results of an investigation into the structural, electrical, and sensor properties of composites obtained under the same conditions using the co-electrospinning method based on ZnO nanofibers modified with 15 mol% MO_x_ (M = Fe^III^, Co^II,III^, Ni^II^, Ga^III^, In^III^, Sn^IV^; [M]/([Zn] + [M]) = 15 mol%) forming complex phases M^I^M^II^O_y_ of *n-* or *p*-type. The difference in the MO_x_-O_ads_ chemisorbed oxygen bond energy and band structure of the MO_x_ component allows for the variation of the electrophysical and sensor properties of nanocomposites. The sensor signal *(S*) was measured depending on the temperature of 60–500 °C toward air pollutants, including reducing the gases CO, methanol, and acetone and oxidizing the gas NO_2_. The temperature corresponding to the maximum sensor signal (T_max_) was compared for different modifiers (MO_x_) as a characteristic of the reactivity of nanofiber heterostructures when interacting with gases.

## 2. Materials and Methods

### 2.1. Materials Synthesis

Nanofibers of ZnO and ZnO/MO_x_ (M = Fe^III^, Co^II,III^, Ni^II^, Ga^III^, In^III^, Sn^IV^) were prepared by co-electrospinning the corresponding salt organic solutions in polyvinylpyrrolidone (PVP, M = 1,300,000) followed by heat treatment of the polymer to remove and metal oxide crystallization. For fiber fabrication, the analytical pure-grade precursors were used (Table 1). The composition of polymer solutions was chosen to reach the same concentration of 15 mol% ([M]/([Zn] + [M])) in all the heterostructures after annealing. The ratio of [M]/([M] + [Zn]) = 15 mol% was chosen based on our previous studies of ZnO/Co composites with different cobalt contents, where this particular composition showed the best properties for use as a sensor material for detecting both oxidizing and reducing gases in a resistance level, sensor response in dry and humid air, and dynamic properties [16]. The electrospinning of polymer solution was carried out at the conditions of 1 mL/h solution feed rate, with a 125 mm distance and 12 kV voltage between the needle and metal collector. The fibrous material was heated at 550 °C (5 h) in air. The annealing conditions for polymer decomposition were determined using thermogravimetric analysis with mass spectral analysis of gaseous products (TG-MS) using a NETZSCH STA 09 PC/PG instrument (Netzsch-Gerätebau GmbH, Selb, Germany).

### 2.2. Materials Characterization

The phase composition of nanofiber was determined with X-ray powder diffraction (XRD) using a DRON-4 diffractometer and Cu Kα radiation (λ = 1.5418 Å). The crystallite size (d_XRD_) was calculated with the Scherrer equation using the full width of half the maximum of the most intense peaks. The chemical composition of samples was studied by X-ray fluorescence (XRF) method using an M1 Mistral X-ray spectrometer (Bruker Nano GmbH, Berlin, Germany). The analyzed area was 1.5 mm, the measurements were carried out at a voltage of 50 kV, and the signal accumulation time was 5 min. Examples of the spectra are shown in Figure S1 (Supporting Information). The results were averaged over three points. The electronic state of modifiers was analyzed using X-ray photoelectron spectroscopy (XPS) using an Axis Ultra DLD (Kratos) spectrometer with an Al K_α_ source. The binding energy was calibrated with a C 1 s peak at 285.0 eV. The XP spectra of the Ga3d, Sn3d, In3d, Co2p, Fe2p, and Ni2p regions and their interpretation are presented in Figure S2 and Table S1, respectively (Supporting Information). The morphology of the nanofibers was studied with scanning electron microscopy (SEM) using a Carl Zeiss NVision 40 electron microscope (Carl Zeiss NTS GmBH, Oberkochen, Germany) at 5 kV. Sample characteristics are summarized in Table 1.

### 2.3. Gas Sensing Measurements

**Figure 1:**
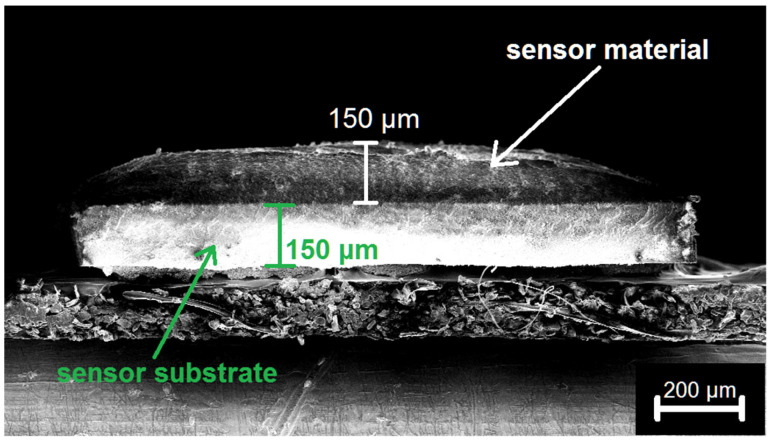
SEM image of thick film formed on alumina substrates.

Thick film (~150 μm, Figure 1) gas sensors of the resistive type based on ZnO/MO_x_ nanofibers were fabricated on alumina substrates embedded in a TO-8 package and provided with Pt electrodes (size 0.3 × 0.2 mm, gap 0.2 mm) and a Pt heater. Powders were dispersed in terpineol and drop-deposited with a micropipette. The sensors were placed in a flow chamber equipped with a PC temperature controller and electrometer. Thick films were heated at 450 °C for 5 h to remove the organic binder.

The sensor properties were investigated using laboratory-made equipment of an original design, which enables the measurement of the resistance in the range of 10^1^–10^11^ Ohms (at a voltage of no more than 1 V) in the controlled temperature range of 20–600 °C. The unit allows the flow rate of the gas mixture to be maintained through the Teflon chamber in the range of 0–300 mL/min and to humidify the gas mixture in the range of RH = 0–90%. DC resistance was measured with the applied voltage 1 V at a temperature range of 60–500 °C. The carrier gas was pure air from a pure air generator model “2,0–3,5” (Himelectronica, Moscow, Russia). The contamination level was within the limits of 10 ppm H_2_O, 2 ppm CO_2_, and 0.1 ppm hydrocarbons. Certified gas mixtures were used as the sources of target gases: 2560 ppm CO, 745 ppm C_3_H_6_O, 1520 ppm CH_3_OH, and 96 ppm NO_2_ (all in N_2_) (Monitoring, Saint-Petersburg, Russia). Gas flows were controlled with mass-flow controllers EL-FLOW (Bronkhorst, Gelderland, the Netherlands). During the exposure of sensors to the background gas, pure air was purged with a flow rate of 100.0 mL/min. The dilution of a target gas to the desired concentration was performed by mixing the flow of a target gas (flow rate 0.1–10 mL/min) with the flow of pure air (flow rate reduced to 99.9–90.0 mL/min). The sensor signal *S* was defined as the ratio of resistance change:

(1) $$S=\frac{R_{g}}{R_{a}}-1\;\text{for the oxidizing gas}{\mathrm{NO}}_{2}$$

(2) $$S=\frac{R_{a}}{R_{g}}-1\;\text{for the reducing gases CO },C_{3}H_{6}O,{\mathrm{CH}}_{3}\mathrm{OH}$$

where *R_a_* is the sensor’s resistance in air, and *R_g_* is the sensor’s resistance in the target gas.

## 3. Results and Discussion

### 3.1. Composition and Microstructure Characterization

It is known [17,18,19] that the sensitivity of thick films of sensor elements can be controlled by both surface reactions and gas diffusion through the sensitive layer. To highlight the role of various modifiers in the formation of the sensor response of zinc oxide-based nanocomposites and to avoid the effect of gas diffusion, we compared the properties of thick film sensors obtained under strictly identical conditions in order to minimize the influence of other characteristics such as film thickness, crystallite size, and specific surface area.

**Figure 2:**
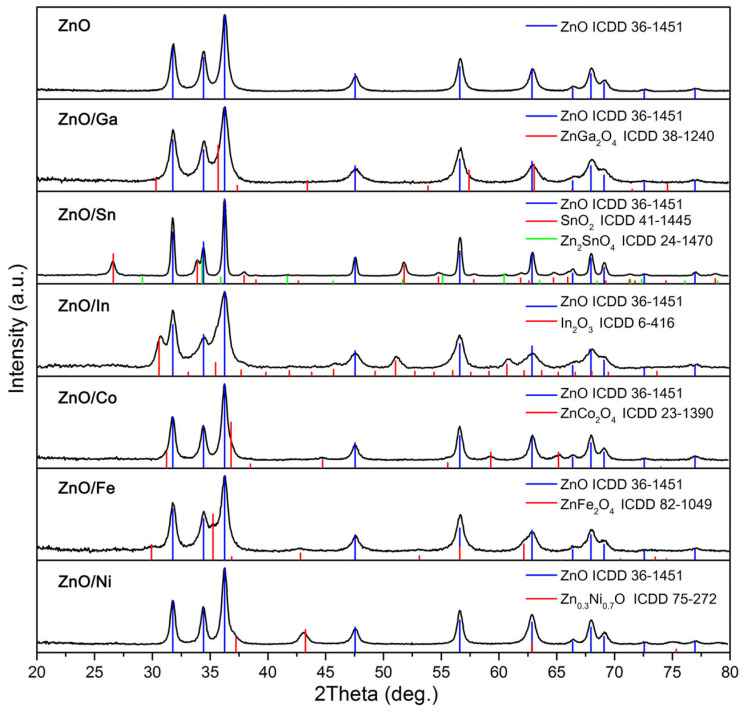
Powder X-ray diffraction patterns of ZnO and ZnO/MO_x_ samples.

The total concentrations of modifiers in ZnO/MO_x_ samples determined with X-ray fluorescence spectroscopy coincide with those set during synthesis within the limits of the analysis error (Table 1). X-ray diffraction data presented in Figure 2 demonstrate that ZnO/MO_x_ samples, except for the ZnO/Sn, contain two crystalline phases (Table 1): zinc oxide with wurtzite structure (ICDD [36-1451]) and the phase of a simple or complex oxide related to MO. The phase composition of the ZnO/Sn sample is more complex and contains three phases: zinc oxide with wurtzite structure (ICDD [36-1451]), tin oxide with rutile structure (ICDD [41-1445]), and zinc orthostannate Zn_2_SnO_4_ (ICDD [24-1470]). The assumption about the presence of the ZnGa_2_O_4_ phase in the ZnO/Ga sample is based on an asymmetric broadening of the (101) wurtzite diffraction maximum (2θ = 36.25°) detected through detailed profile analysis of ZnO and ZnO/Ga diffraction patterns (Figures S3 and S4, Supporting Information).

**Figure 3:**
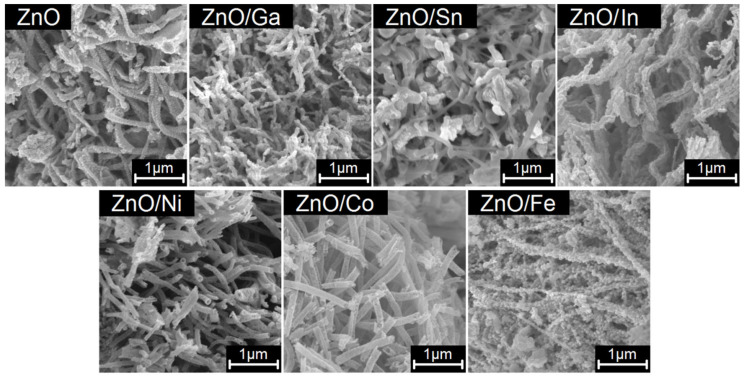
SEM images of ZnO and ZnO/MO_x_ samples.

According to the scanning electron microscopy (SEM) data, all samples are three-dimensional, highly porous spongy structures consisting of polycrystalline nanofibers longer than 10 μm (Figure 3). Fibers of ZnO/In, ZnO/Co, and ZnO/Ni heterostructures form tubes with a diameter of 80–100 nm that may be caused by the difference in the rates of crystallization of ZnO and MO_x_ oxides and mutual diffusion of components. From the low-temperature nitrogen adsorption data, the specific surface area of all samples is about 13–15 m^2^/g.

### 3.2. Electrical Conductivity Measurements

**Figure 4:**
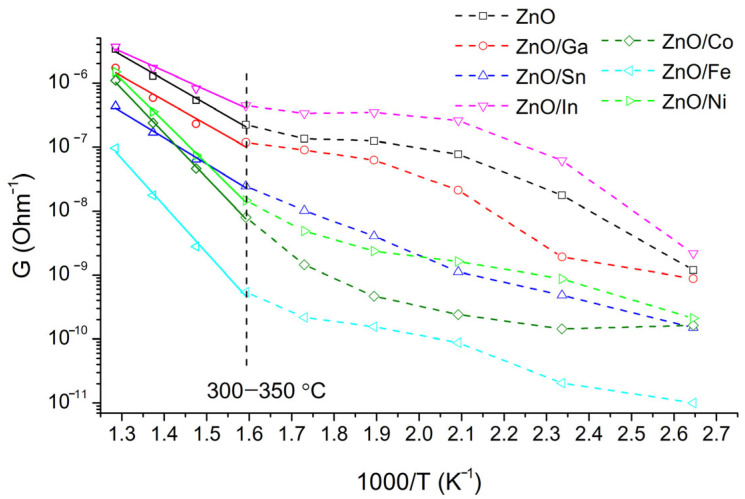
Temperature dependences of ZnO/MO_x_ conductivity in dry air.

Figure 4 shows the temperature dependence of the ZnO/MO_x_ conductivity. All measurements were carried out with a stepwise decrease in temperature. The resistance of the ZnO/Co, ZnO/Fe, and ZnO/Ni samples in the entire temperature range is 2–3 orders of magnitude higher relative to the ZnO, ZnO/Ga, ZnO/Sn, and ZnO/In samples that may be associated with the formation of a significant barrier at the grain interface in samples ZnO/Co, ZnO/Fe, and ZnO/Ni. Formed at the interface complex oxides containing modifier cations: ZnCo_2_O_4_ (work function *φ* = 5.6 eV [20], ZnFe_2_O_4_ (*φ* = 5.46 eV [21]), and Zn_x_Ni_1-x_O (*φ* = 6.3 eV for NiO [22]) have *p*-type conductivity, while ZnO (*φ* = 5.2 eV [23]), ZnGa_2_O_4_ (*φ* = 3.54 eV [24]), SnO_2_ (*φ* = 4.7 eV [25]), and In_2_O_3_ (*φ* = 4.3 eV [26]) are characterized by *n*-type conductivity. The high conductivity of ZnO/Ga, ZnO/Sn, and ZnO/In heterostructures can be due to the electron accumulation layer formed in ZnO grains and the effect of doping zinc oxide, for which In, Ga, and Sn are the effective donor impurities. At low temperatures of 100–300 °C, the conductivity behavior is complex and does not linearize in the lnG-(1/T) coordinates due to oxygen adsorption/desorption on the surface of semiconductor oxides. These processes are accompanied by the capture and delocalization of electrons in the near-surface layer and affect the conductivity of the materials. Additionally, a comparison of the temperature dependences of ZnO/MO_x_ electrical conductivity in dry air and in dry nitrogen is shown in Figure S5 (Supporting Information). The electrical conductivity of materials in dry nitrogen always exceeds the electrical conductivity in dry air. It can be noted that this difference tends to increase with decreasing temperature, which indicates an increased contribution of oxygen adsorption on the semiconductor surface in the low-temperature region.

In the range of 350–500 °C, the temperature dependences of the conductivity are linearized in lnG-(1/T) coordinates, which indicates that conductivity has an activation mechanism. The activation energy E_a_ of conductivity for ZnO/Co, ZnO/Fe, and ZnO/Ni composites with *p-n* heterojunctions is higher than that for ZnO/Ga, ZnO/Sn, and ZnO/In samples with *n-n* heterojunction and pure ZnO (Table 2). This effect can be explained using a model of an inhomogeneous semiconductor with large-scale potential fluctuations, which consists of a smooth band’s bending with the formation of a random potential barrier caused by the potential of charged impurities [27]. According to this model, the movement of an electron is impossible in a modulated zone if its energy is below a certain level necessary to overcome barriers (mobility threshold level), which is determined by the nature of the potential relief. Thus, the energy determined from the temperature dependences of the conductivity is the energy necessary for the carrier’s activation from the Fermi level to the mobility threshold level. The increase in the activation energy of conductivity for composites with *p-n* heterocontacts is probably due to the formation of a larger potential barrier at the *p-n* ZnO/MO_x_ interface compared to *n-n* heterostructures. This leads to a larger-scale modulation of the band’s relief that increases the mobility threshold level and, accordingly, the activation energy of the conductivity.

### 3.3. Gas Sensor Measurements

#### 3.3.1. Detection of Reducing Gases

**Figure 5:**
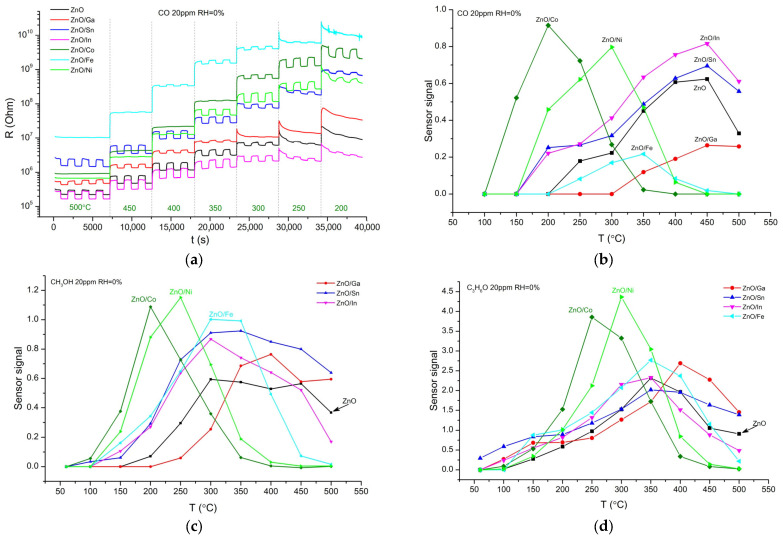
Dynamic change of the resistance of ZnO/MO_x_ nanofibers when detecting 20 ppm CO in dry air at different working temperatures (**a**). Temperature dependences of the sensor response of ZnO/MO_x_ nanofibers when detecting 20 ppm CO (**b**), 20 ppm methanol (**c**), and 20 ppm acetone (**d**) in dry air.

The sensor properties of ZnO/MO_x_ nanofibers were studied when detecting the reducing gases CO (20 ppm), acetone (20 ppm), and methanol (20 ppm) in the temperature range of 60–500 °C. The dynamic response of the material resistance to the introduction of 20 ppm of CO into background dry air depending on temperature is shown in Figure 5a. As illustrated in Figure 5b–d, ZnO/MO_x_ nanofibers are characterized by different temperature dependences of the sensor signal. When detecting reducing gases, *p-n* heterostructures ZnO/Co and ZnO/Ni demonstrate maximum sensor response at lower temperatures compared to ZnO and *n-n* heterostructures ZnO/In, ZnO/Ga and ZnO/Sn.

Differences in the sensor response of *n*-type and *p*-type semiconductor oxides, as well as nanocomposites with *p-n* heterojunctions, due to different mechanisms of electrical conductivity, are discussed in [10,28,29], respectively. The main parameters responsible for the sensor response value are the initial band bending, which is determined by the ratio of the concentration of charge carriers near the surface and in the bulk of crystallites, as well as morphological parameter determined by the average grain size and the contact size [29]. Taking into account that the morphological parameter does not differ for the considered ZnO/MOx nanofibers, it can be assumed that the sensor response is determined by the initial band bending and its change depending on the temperature and surface reactivity of *n*-type and *p*-type components when interacting with oxygen.

**Figure 6:**
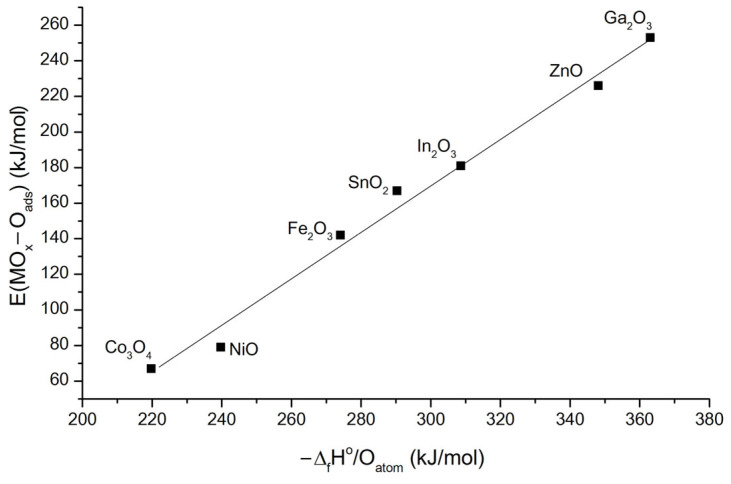
Correlation of the binding energy of adsorbed oxygen with the surface of the oxide E_MOx_-O_ads_ and the energy of oxide formation attributed to the number of oxygen atoms in the oxide −Δ_f_H°/O_atom_ [1,31].

**Figure 7:**
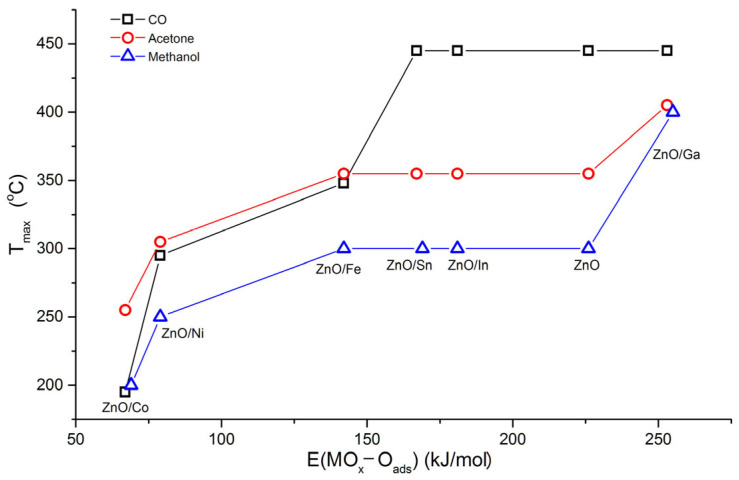
Temperature of the maximum sensor signal T_max_ of ZnO/MO_x_ nanofibers when detecting CO, acetone, and methanol (all 20 ppm in dry air) on the binding energy of chemisorbed oxygen for binary metal oxides containing modifier cations.

The difference in the temperature of the maximum sensor response T_max_ when detecting reducing gases can be discussed based on the binding energies of chemisorbed oxygen E(MO-O_ads_). The binding energy between the surface of some binary metal oxides and chemisorbed oxygen is shown in Figure 6, according to Boreskov’s works [30]. Figure 7 demonstrates for ZnO/MO_x_ nanofibers the dependences of the temperature of the maximum sensor signal T_max_ toward CO, acetone, and methanol on the binding energy of chemisorbed oxygen for binary metal oxides containing modifier cations.

In the case of methanol and acetone, in general, one can note a tendency to increase T_max_ with an increase in E(MO-O_ads_). In particular, Co_3_O_4_ and NiO are characterized by the weakest binding energy of chemisorbed oxygen with the oxide’s surface that, for ZnO/Co and ZnO/Ni samples, can explain high sensor signal and low T_max_ when interacting with reducing gases by the following reaction:

(3) $$\beta \cdot R_{\left(gas\right)}+O_{\beta \left(ads\right)}^{\alpha -}\to \beta \cdot {RO}_{\left(gas\right)}+\alpha \cdot e^{-},$$

where $O_{\beta \left(ads\right)}^{\alpha -}$ is chemisorbed oxygen species, *RO*_(*gas*)_ is the product of the oxidation of reducing gas *R*_(*gas*)_, and *e^−^* is a free electron.

The values of the maximum sensor response of ZnO and *n-n* heterostructures ZnO/Ga, ZnO/In, and ZnO/Sn toward methanol (Figure 5c) demonstrate an upward trend with an increase in the Lewis acidity A_L_ of oxides in the raw ZnO (A_L_ = 1.6) < Ga_2_O_3_ (A_L_ = 2.5) < In_2_O_3_ (A_L_ = 2.6) < SnO_2_ (A_L_ = 3.5) [32]. It can be assumed that Lewis acid sites at the surface of semiconductor metal oxides serve as adsorption sites for CH_3_OH molecules and, thus, favor the oxidation of target molecules at appropriate temperatures [1].

In the case of acetone detection (Figure 5d), no correlation was observed between sensor signals and Lewis acidity of ZnO and *n-n* heterostructures. It suggests that the determining factor for the sensor response should be the reactivity of the materials surface in oxidation Reaction (3) [2]. The difference in the sensor response values may be due, among other things, to the inequality in the predominant form of chemisorbed oxygen $O_{\beta \left(ads\right)}^{\alpha -}$, which at a fixed temperature is determined by the composition and microstructure parameters of the semiconductor oxide [33].

When detecting CO, ZnO/MO_x_ nanofibers can be divided into two groups. For composites with *p-n* heterojunctions, T_max_ increases with E(MO-O_ads_), while for ZnO and composites with *n-n* heterojunctions, the temperature of the maximum sensor signal is much higher (about 400–450 °C) and practically does not depend on the binding energy of chemisorbed oxygen. This effect becomes clear if one takes into account that the sensor response of unmodified ZnO when detecting CO in dry air is due to CO oxidation by chemisorbed oxygen $O_{\beta \left(ads\right)}^{\alpha -}$ (Reaction (3)) and lattice oxygen $O_{lat}$ (Reaction (4)) (Mars–van Krevelen mechanism) [34]:

(4) $${CO}_{\left(gas\right)}+O_{lat}\to {CO}_{2\left(gas\right)}+V_{O}^{2+}+2e^{-},$$

where $V_{O}^{2+}$ is the doubly ionized oxygen vacancy in the ZnO lattice.

Since ZnO is the dominant phase in ZnO/MO_x_ nanofibers, and the modifier does not possess a sufficiently low binding energy with chemisorbed oxygen, it can be assumed that the main contribution to the sensor response is made by the CO oxidation by ZnO lattice oxygen that determines such a high T_max_ value.

#### 3.3.2. Detection of Oxidizing Gases

The presence of NO_2_ in dry air leads to an increase in the resistance of all materials because NO_2_ molecules are electron acceptors and act as electron traps upon adsorption on the metal oxide surface. The mechanism of NO_2_ detection is based on ionosorption of analyte molecules to form surface nitrites ${NO}_{2(ads)}^{-}$:

(5) $${NO}_{2(gas)}+e^{-}\to {NO}_{2(ads)}^{-}$$

which undergoes disproportionation and/or conversion, resulting in ${NO}_{3(ads)}^{-},$ ${NO}_{\left(ads\right)}^{+}$, etc. [2]. The authors of [35] showed using Raman spectroscopy that the electrical conductivity of *n*-type SnO_2_ in the presence of NO_2_ correlates with the concentration of surface bidentate nitrite groups. Ab initio calculations [36] indicate that the most energetically advantageous is the molecular adsorption of nitrogen dioxide with the coordination of one or both oxygen atoms of the NO_2_ molecule on the vacancies of bridging oxygen atoms of the partially reduced tin dioxide surface. IR spectroscopy and programmable thermal desorption in the temperature range of 25–400 °C [37] have shown the possibility of NO_2_ dissociation upon interaction with the surface of tin dioxide. The presence of NO molecules in the thermal desorption products may serve as indirect evidence of the following reaction:

(6) $${NO}_{2(ads)}\to {NO}_{\left(gas\right)}+O_{\left(ads\right)}^{-}$$

The typical sites for NO_2_ adsorption on the ZnO surface are oxygen vacancies, on which, according to XPS and IR studies, near room temperature, a NO_2_ molecule can orient itself in different ways, with the formation of NO_2_^−^ and NO_3_^−^ ions [38,39,40,41,42,43,44]. In situ mass spectrometry study of the photostimulated interaction of O_2_ and NO_2_ molecules with the ZnO surface under UV radiation in the temperature range of 30–100 °C demonstrated that the sensor response to NO_2_ requires both unoccupied adsorption sites and free electrons that can be expressed by the following equations [45]:

(7) $${NO}_{2(gas)}+V_{O}+e^{-}\to {NO}_{2}^{-}\ldots V_{O}$$

(8) $${NO}_{2(gas)}+O_{lat}+V_{O}+e^{-}\to {NO}_{3}^{-}\ldots V_{O}$$

**Figure 8:**
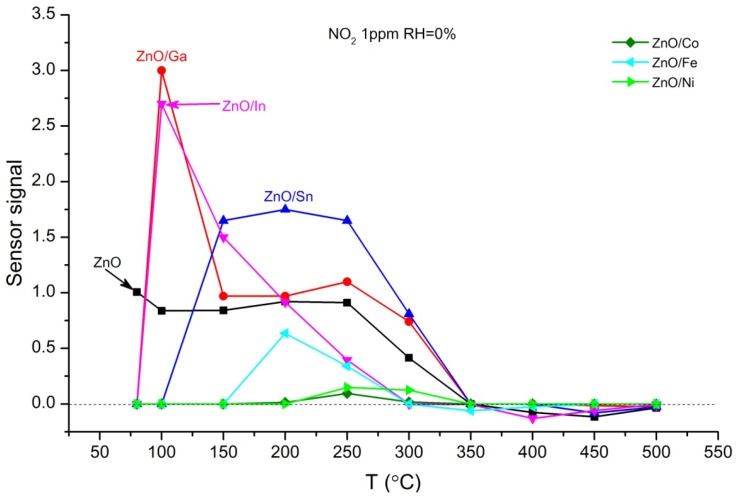
Temperature dependences of the sensor response of ZnO/MO_x_ nanofibers when detecting 1 ppm NO_2_ in dry air.

Figure 8 shows the temperature dependences of the sensor signal *S* of ZnO and ZnO/MO_x_ nanofibers toward 1 ppm NO_2_ in dry air in the temperature range of 80–500 °C. The ZnO/Ga and ZnO/In composites demonstrate the highest sensor signal at a temperature of 100 °C that may be associated with the doping effect of Ga^3+^ and In^3+^ cations in ZnO leading to an increase in the concentration of donor defects—oxygen vacancies and free electrons favoring Reaction (5) [2,46].

**Figure 9:**
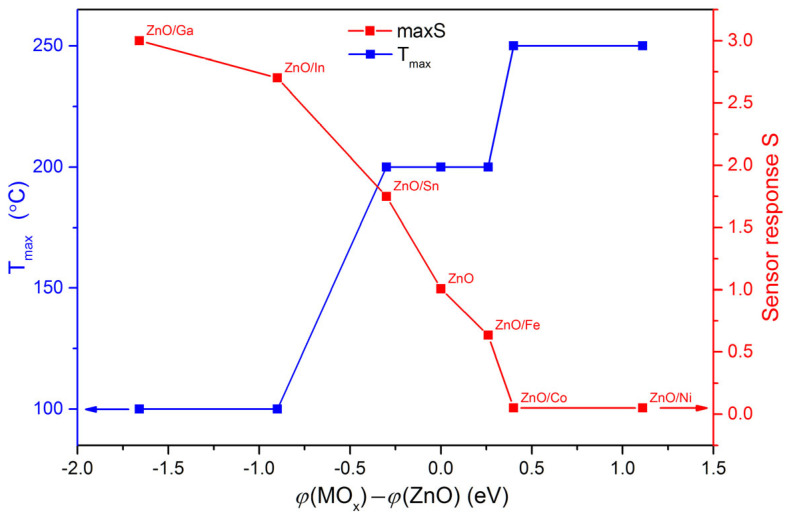
Maximum sensor response S and temperature of the maximum sensor signal T_max_ of ZnO/MO_x_ nanofibers when detecting 1 ppm NO_2_ in dry air vs. the band bending in ZnO/MO_x_ nanofibers.

Since the formation of the sensor response of semiconductor metal oxides toward oxidizing gases is due to the localization of free electrons and, accordingly, depends on the concentration of electrons in the near-surface layer, the maximum sensor signal *S* and the temperature of the maximum sensor signal T_max_ may depend on the potential barrier at the interface between ZnO and a complex oxide containing a modifier. The band bending at the ZnO/MO_x_ interface can be characterized by the difference in the electron work function as *φ*(MO) − *φ*(ZnO). The dependence of S and T_max_ values on band bending in ZnO/MO_x_ nanofibers is shown in Figure 9.

It follows from Figure 9 that the maximum sensor response S, as well as the temperature of the maximum sensor signal T_max_ when detecting 1 ppm NO_2_ in dry air, monotonously, but oppositely directed, changes with the band bending in ZnO/MO_x_ nanofibers. Thus, as the band bending decreases, the sensor signal increases, and the temperature of the maximum sensor signal decreases. The formation of a heterocontact between ZnO and a semiconductor with a larger work function leads to the electron depletion of the near-surface of ZnO grains, which causes a decrease in the sensor response of the composite. On the contrary, when forming a heterocontact between ZnO and a semiconductor with a smaller work function, the concentration of electrons in the near-surface layer of ZnO grains increases, providing an increase in sensor response. The temperature corresponding to the maximum sensor signal of ZnO/MO_x_ nanocomposites toward NO_2_ correlates with the value of the activation energy of conductivity, discussed in Section 3.2 (Table 2). In the case of composites with a *p-n* heterocontact, a higher temperature is necessary for the electrons to overcome the activation barrier of conductivity and form a significant electrical response.

## 4. Conclusions

The introduction of M = Fe^III^, Co^II,III^, Ni^II^, Sn^IV^, In^III^, and Ga^III^ modifiers into ZnO nanofibers in the co-electrospinning synthesis leads to the formation of multiphase ZnO/MO_x_ nanocomposites containing ZnO with a wurtzite structure and a complex oxide including modifier cations. The study of the sensor properties of ZnO and ZnO/MO_x_ nanofibers when detecting reducing gases CO, methanol, and acetone showed that low binding energy of chemisorbed oxygen with the surface of the modifier’s oxides determines the high sensitivity of materials toward reducing gases in the low temperature range of 200–300 °C. When detecting oxidizing gases, for example, NO_2_, the most important condition for achieving high sensitivity is a high concentration of donor active sites and free electrons in the near-surface layer, which is associated with the band bending at the ZnO/MO_x_ interface characterized by the difference in the electron work function of ZnO and MO_x_. Nanocomposites ZnO/Ga, ZnO/In, and Zn/Sn, in which the electron work function of the MO_x_ is smaller than the corresponding value for zinc oxide, demonstrate high sensitivity toward NO_2_ at temperatures of 100–200 °C.

## Figures and Tables

**Table 1:** Composition and microstructure parameters of ZnO and ZnO/MO_x_ nanofibers.

Sample	MO_x_ Precursor	M/(Zn + M) ^1^, mol% (XRF)	Phase Composition (XRD)	d_XRD_ ^2^, nm	Morphology
ZnO	-	-	ZnO (wurtzite)	14 ± 1	fibers
ZnO/Co	Co(CH_3_COO)_2_∙4H_2_O	15.3 ± 0.8	ZnO (wurtzite); Zn_x_Co_3-x_O_4_ (cubic)	15 ± 1 12 ± 1	tubes
ZnO/Ni	Ni(NO_3_)_2_∙6H_2_O	16 ± 1	ZnO (wurtzite); Zn_x_Ni_1-x_O (cubic)	16 ± 1 10 ± 1	tubes
ZnO/Fe	Fe(acac)_3_	15.0 ± 0.9	ZnO (wurtzite); ZnFe_2_O_4_ (cubic)	11 ± 1 9 ± 1	fibers
ZnO/Sn	SnCl_4_∙5H_2_O	14.3 ± 0.8	ZnO (wurtzite); SnO_2_ (tetragonal); Zn_2_SnO_4_ (cubic)	26 ± 2 13 ± 1 -	fibers
ZnO/In	In(NO_3_)_3_∙4.5H_2_O	14.8 ± 0.8	ZnO (wurtzite); In_2_O_3_ (cubic)	10 ± 1 10 ± 1	tubes
ZnO/Ga	Ga(NO_3_)_3_∙9H_2_O	14.6 ± 0.9	ZnO (wurtzite); ZnGa_2_O_4_ (cubic)	10 ± 1 9 ± 1	fibers

^1^ Content of the second component determined with X-ray fluorescence analysis. ^2^ Size of the coherent scattering region determined from the broadening of diffraction peaks using Scherrer equation.

**Table 2:** Activation energy of conductivity (E_a_) and difference in the electron work functions *φ*(MO) − *φ*(ZnO) for ZnO and ZnO/MO_x_ nanofibers.

Sample	MO_x_ Phase ^1^	E_a_, eV (T = 350–500 °C)	*φ*(MO) − *φ*(ZnO), eV
ZnO	-	0.46	-
ZnO/Co	ZnCo_2_O_4_	0.84	0.4
ZnO/Ni	NiO	0.79	1.1
ZnO/Fe	ZnFe_2_O_4_	0.89	0.26
ZnO/Sn	SnO_2_	0.49	−0.5
ZnO/In	In_2_O_3_	0.36	−0.9
ZnO/Ga	ZnGa_2_O_4_	0.45	−1.66

^1^ The work function value *φ* for this phase is used.

## Data Availability

The original contributions presented in the study are included in the article. Further inquiries can be directed to the corresponding author.

## Supplementary Materials

The following supporting information can be downloaded at: https://www.mdpi.com/article/10.3390/s25020376/s1, Figure S1. XRF spectra of unmodified ZnO and ZnO/MOx nanofibers; Figure S2. XP spectra of modifiers in ZnO/MO_x_; Figure S3. XRD patterns of ZnO and ZnO/Ga showing the presence of the ZnGa_2_O_4_ phase in ZnO/Ga; Figure S4. Profile analysis of ZnO/Ga diffraction pattern sample without (a) and with additional reflection from ZnGa_2_O_4_ phase (b); Figure S5. Comparison of the temperature dependences of ZnO/MOx electrical conductivity in dry air and in dry nitrogen; Table S1. Interpretation of XP spectra of ZnO/MOx nanofibers.
