# Supplementary material for: ZnO/MOx Nanofiber Heterostructures: MOx Receptor’s Role in Gas Detection
Source: Sensors (Basel). 2025 Jan 10;25(2):376. doi: 10.3390/s25020376 (PMC11768764; doi:10.3390/s25020376)
Supplement: Supplementary file 1 [file sensors-25-00376-s001.zip › sensors-3385533-supplementary.pdf]

Supporting Information

ZnO/MO_x nanofiber heterostructures: MO_x receptor role in gas detection

Vadim Platonov [1], Oleg Sinyashin [2] and Marina Rumyantseva [1,*]

[1] Chemistry Department, Moscow State University, Moscow, 119991 Russia; E-mails: agnes1992@yandex.ru (V.P.), roum@inorg.chem.msu.ru (M.R.)

[2] Federal Research Center Kazan Scientific Center RAS, Kazan, 420111 Russia; buran0071@yandex.ru

* Correspondence: roum@inorg.chem.msu.ru; Tel.: +7-495-939-5471

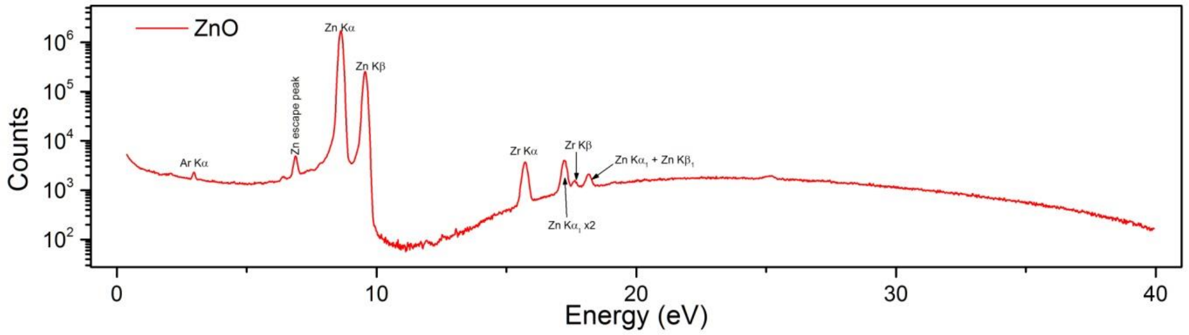

Figure S1a. XRF spectrum of unmodified ZnO

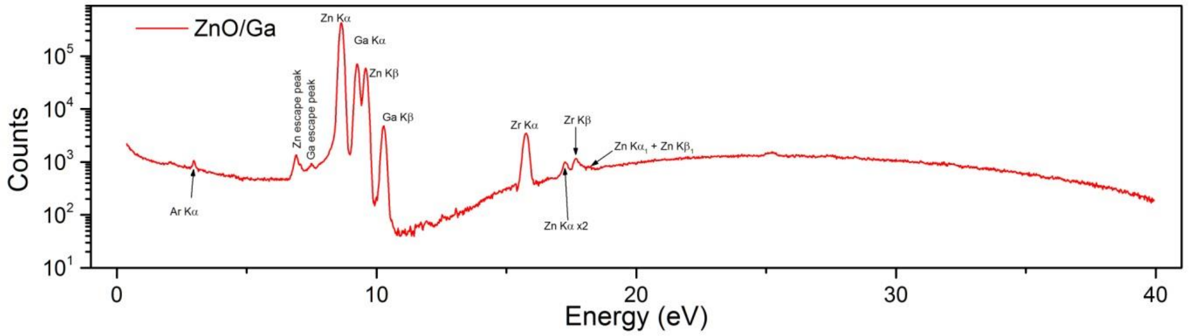

Figure S1b. XRF spectrum of ZnO/Ga sample

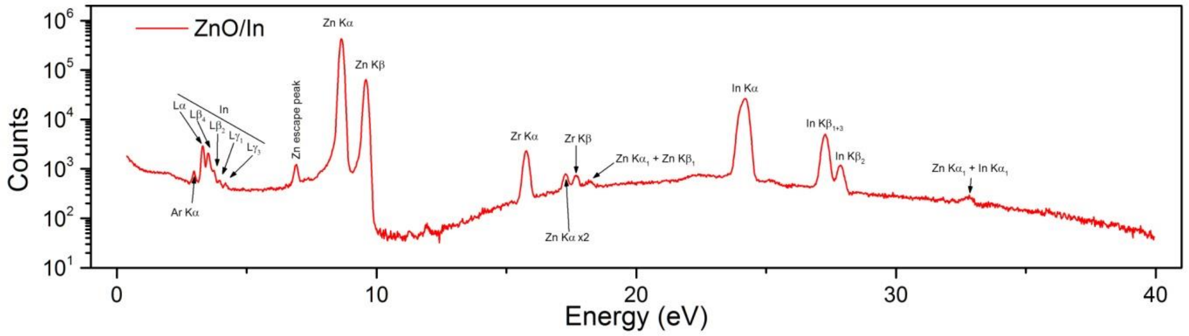

Figure S1c. XRF spectrum of ZnO/In sample

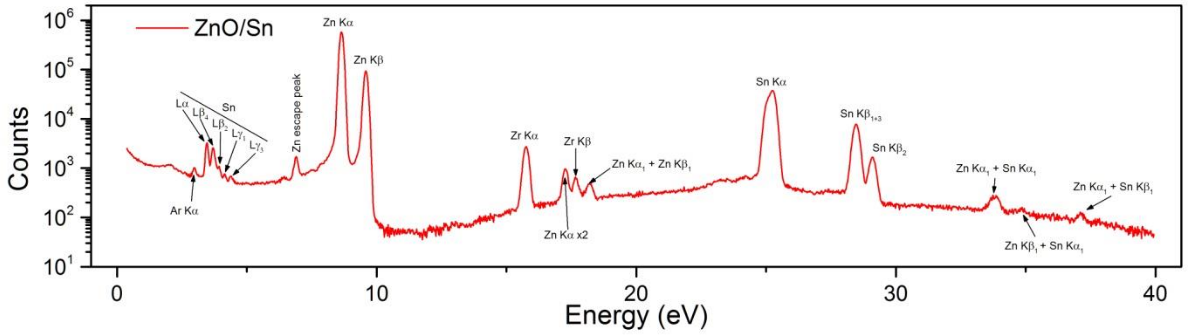

Figure S1d. XRF spectrum of ZnO/Sn sample

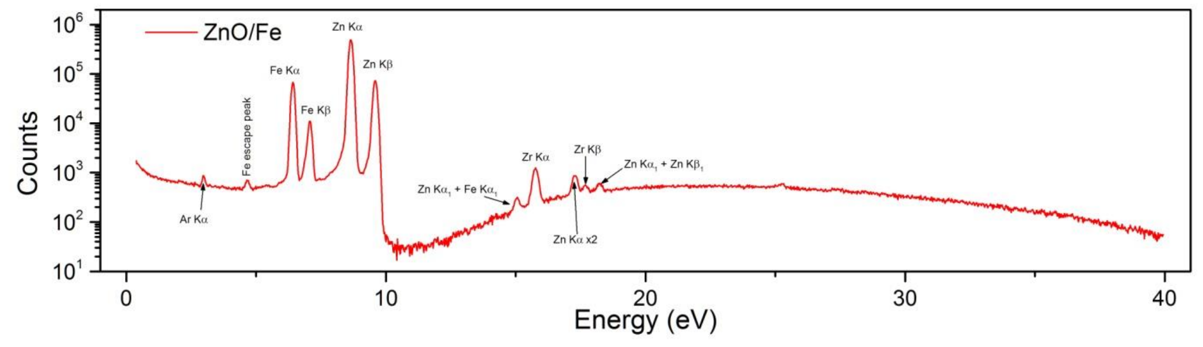

Figure S1e. XRF spectrum of ZnO/Fe sample

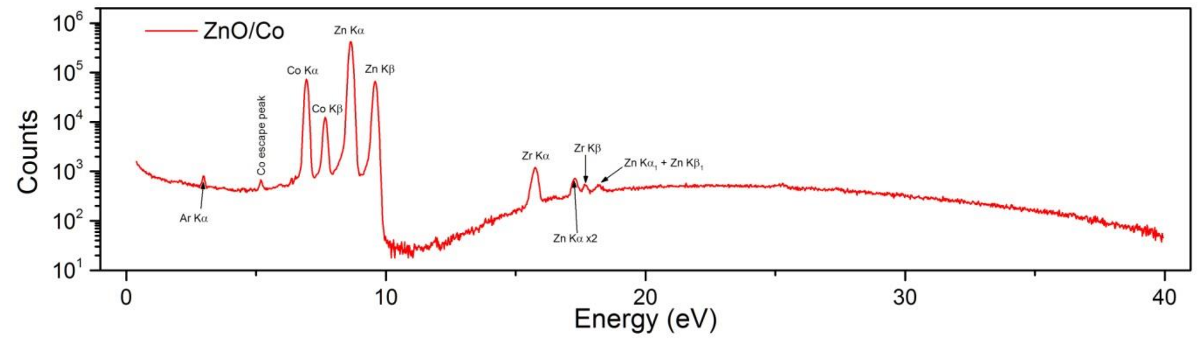

Figure S1f. XRF spectrum of ZnO/Co sample

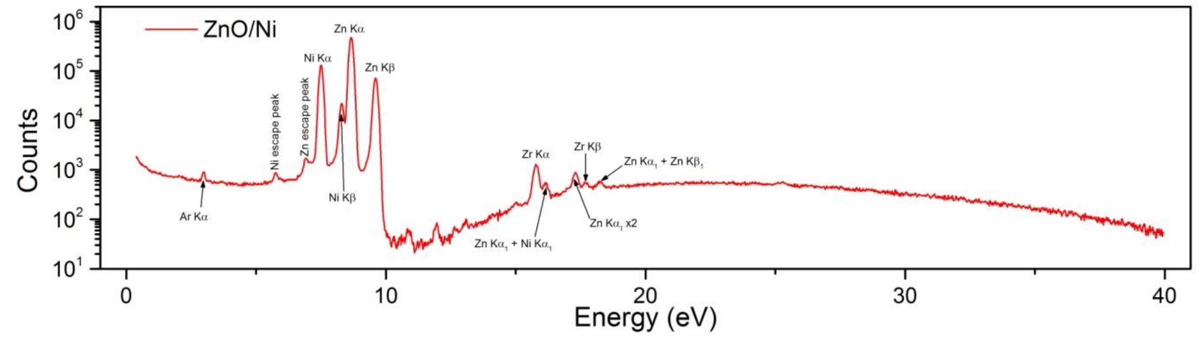

Figure S1g. XRF spectrum of ZnO/Ni sample

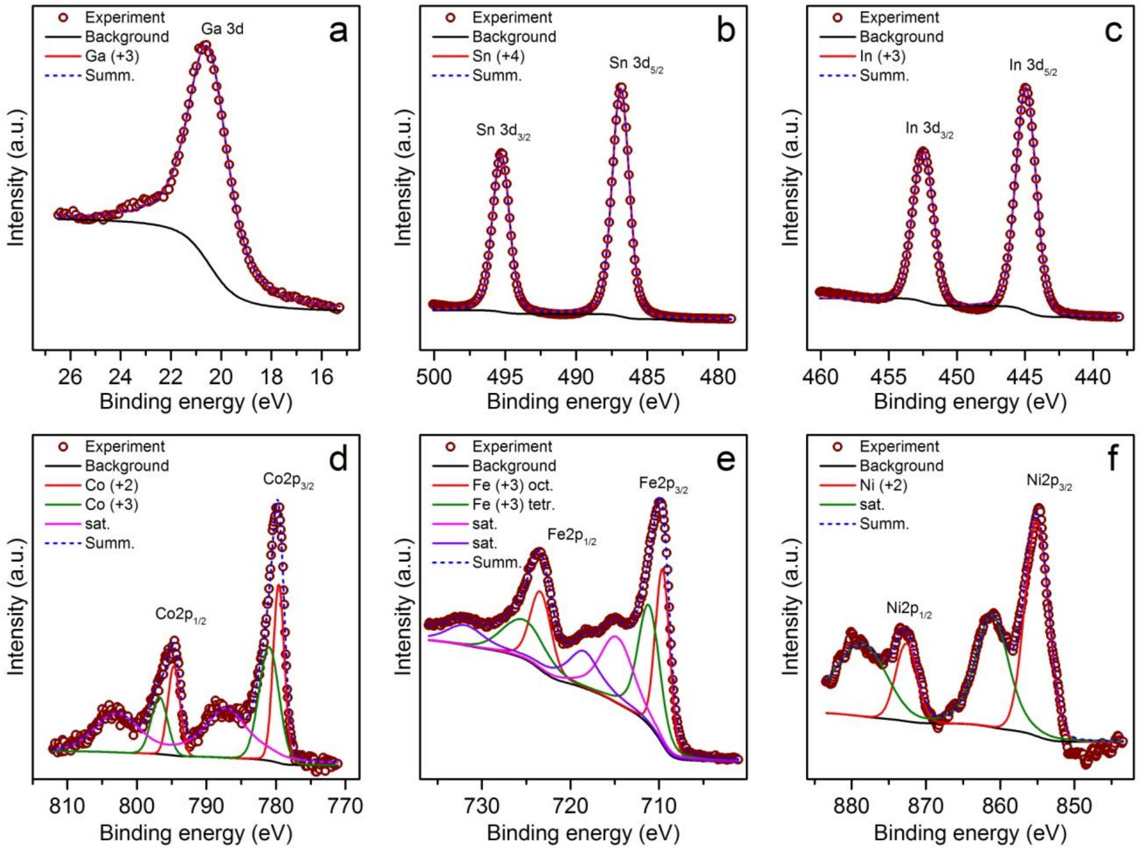

Figure S2. XP spectra of modifiers in ZnO/MOx.

Table S1. Interpretation of XP-spectra of ZnO/MOx nanofibers

**Sample**	**E, eV**		**Modifier oxidation state**	**Crystalline phase**
ZnO/Ga	20.6		Ga^{2+}	$ZnGa_2O_4$
ZnO/In	445.0	452.5	In^{3+}	In_2O_3
ZnO/Sn	486.9	495.3	Sn^{4+}	SnO_2, Zn_2SnO_4
ZnO/Fe	709.6	723.5	Fe^{3+} (oct.)	$ZnFe_2O_4$
	711.2	725.7	Fe^{3+} (tetr.)	
ZnO/Ni	855.2	872.7	Ni^{2+}	$Zn_xNi_{1-x}O$
ZnO/Co	779.6	795.7	Co^{3+}	$Zn_xCo_{3-x}O_4$
	781.0	796.8	Co^{2+}	

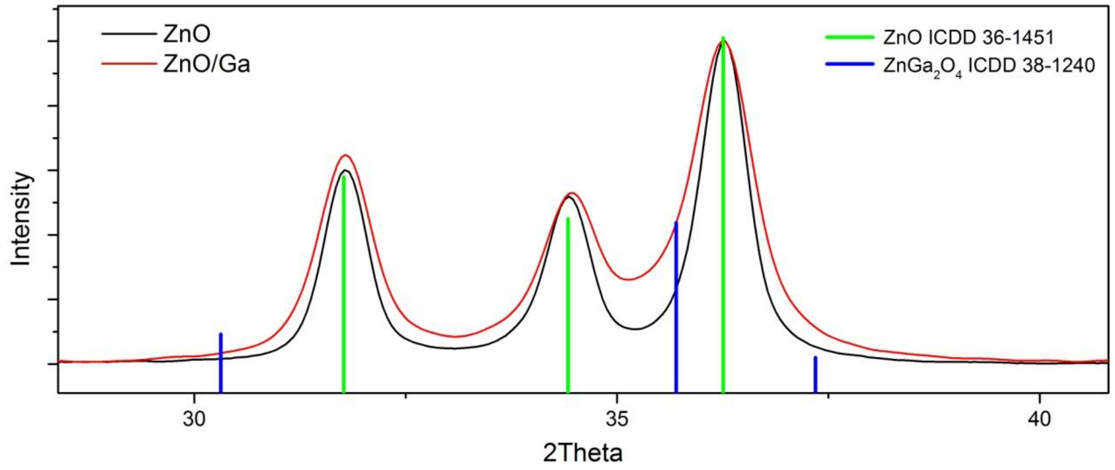

Figure S3. XRD patterns of ZnO and ZnO/Ga showing the presence of the phase $ZnGa_2O_4$ in ZnO/Ga.

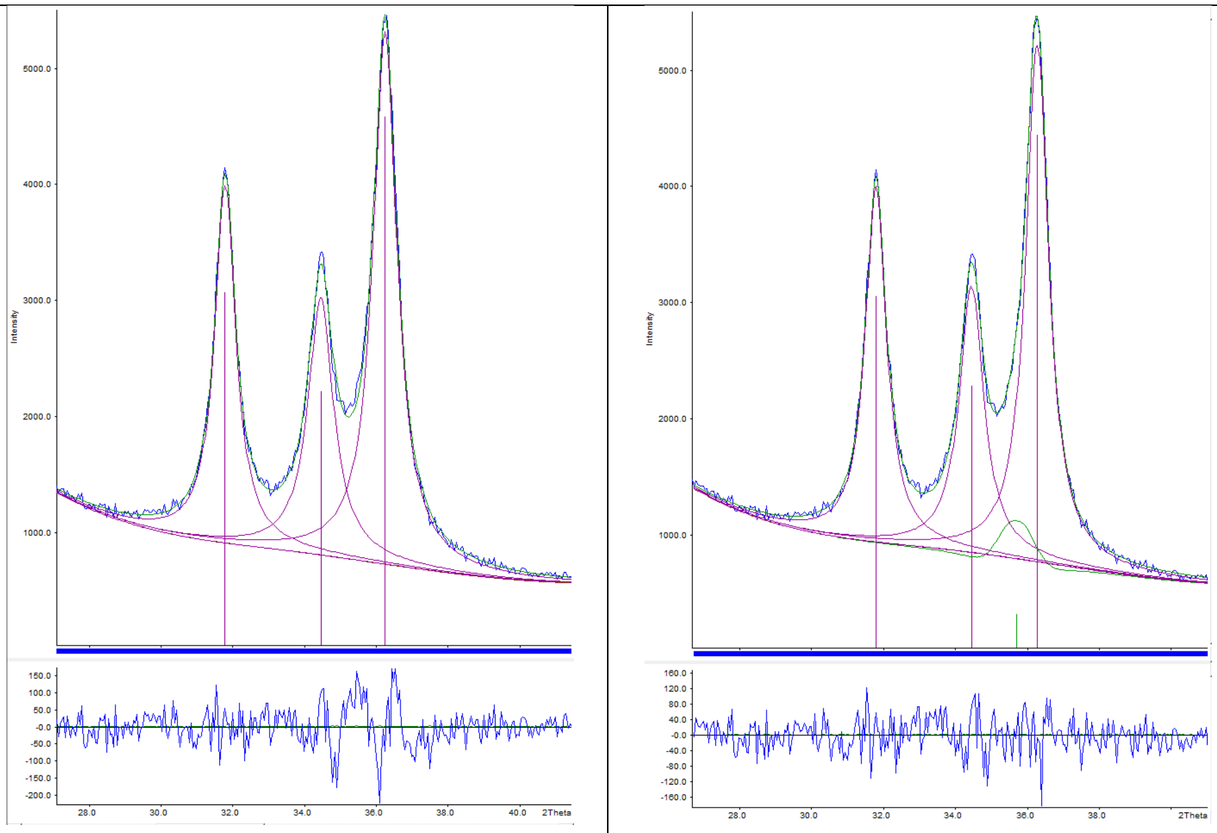

(a)	(b)

Figure S4. Profile analysis of ZnO/Ga diffraction pattern sample without (a) and with additional reflection from $ZnGa_2O_4$ phase(b)

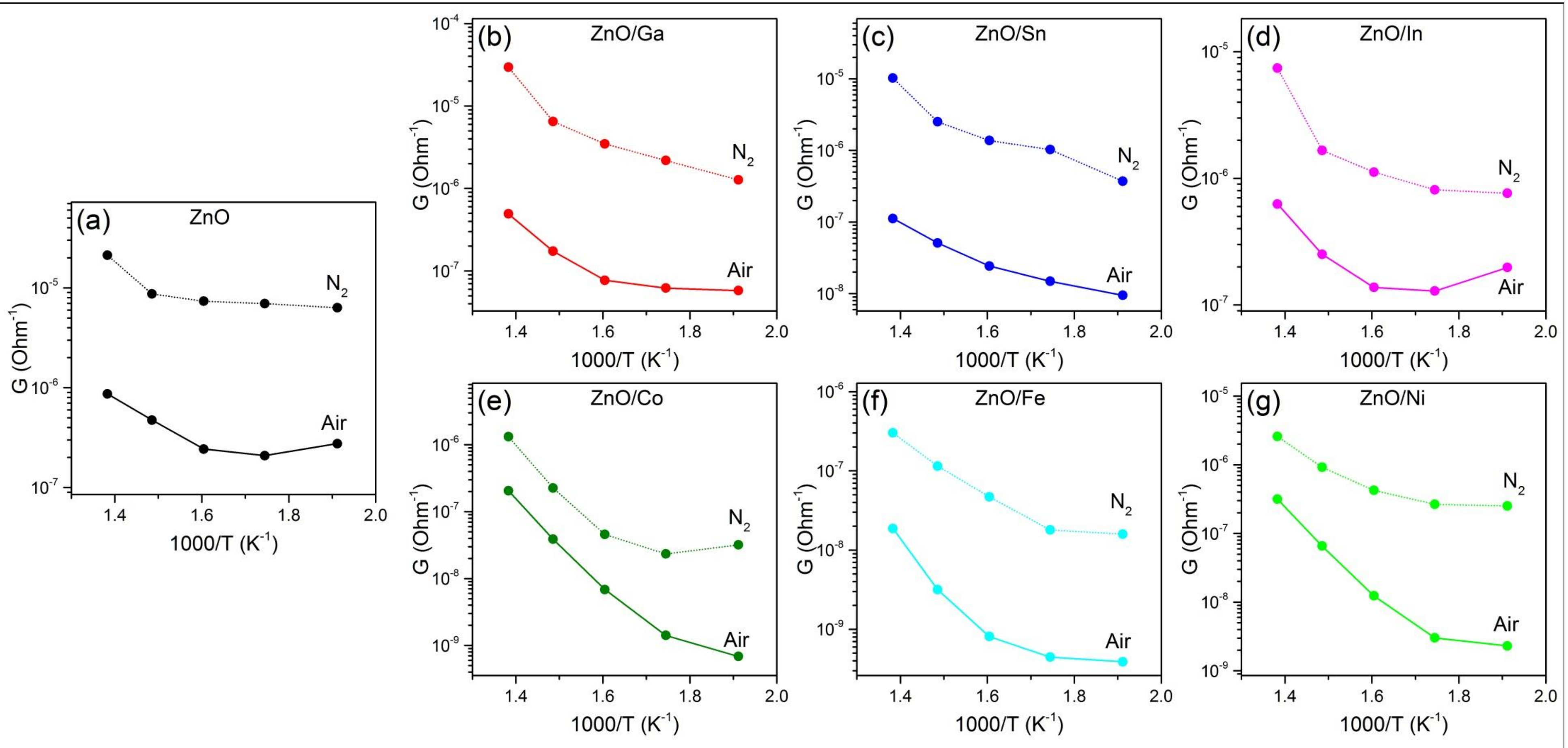

Figure S5. Comparison of the temperature dependences of ZnO/MOx electrical conductivity in dry air and in dry nitrogen
